# 

**Published:** 1892-09-10

**Authors:** 


**Notices of Births, Deaths, and Marriages,** are inserted in this column at a charge of 2s. 6d. for an announcement not exceeding four lines.

### NOTICE TO CORRESPONDENTS.

All MS., Letters, Books for Review, and other matters intended for the Editor should be addressed The Editor, The Lodge, Porchester Square, London, W.

The Editor cannot undertake to return rejected M.S., even when accompanied by stamped directed envelope.

**Notice to Advertisers and Subscribers.**

All Advertisements, Orders for Copies of The Hospital, and Business Communications should be addressed to The Publisher, *not to the Editor*, The Hospital, 140, Strand, W.C.
The Cost of Subscription, post free, is as follows:—Per week, 2½d.; per quarter, 2s. 6d.; per half-year, 5s.; per annum, 8s. 6d. Foreign:—Per annum, 10s. 8d.; payable, in all cases, in advance.

**"Burdett's Hospital Annual," 1891—1892.**

Third year of publication. 600 pp. Boards, gilt lettering. A most complete guide to British and Colonial Hospitals, Dispensaries, Nursing and Convalescent Institutions, and Asylums. Price 3s. 6d. Published by The Scientific Press (Limited), 140, Strand, W.C.

**NOTICE.—The Index for Vol. XI. (ending March, 1892) is on sale at the Office of this Journal, price 2½d., post free.**

### Scraps and Gleanings.

The total sum collected on Hospital Saturday at Folkestone amounted to £102 0s. 5¼d.

The annual Saturday collection in aid of the Tyrrell Cottage Hospital at Ilfracombe, was made on Saturday, 27th. The total amount cleared was £54 19s. 3d.

The promoters of the Kenilworth Hospital Saturday movement hope to forward a cheque for £46 to the Warneford Hospital, as the result of this year's collection.

Two new consulting rooms are now being added to the Edinburgh Royal Dispensary, and a number of alterations are also being made. The total cost of the work, the plans for which were prepared by Mr. R. Wilson, architect, is close upon £400.

According to the report of the Flower Girls' Mission, the trade in cut flowers in London amounts to £5,000 a-day. Although this includes the profit of the florists, a large amount of this sum still remains to be accounted for by the earnings of the flower girls.

The quarterly report of the Belfast Royal Hospital shows that during the previous three months 690 intern and 5,731 extern cases had been treated. All accounts against the hospital up to the end of July last had been paid, the result being due to the centenary bazaar.

Baron Hirsch has, through his trainer, Mr. John Porter, of the Park House Racing Stables at Kingsclere, expressed his willingness to erect a cottage hospital in that place, to signalize the successes achieved by his horses during the period they have been under Mr. Porter's care.

The objects of the Metropolitan Hospital Aid League will be to raise in the readiest and best possible way a large annual fund in aid of the London medical charities; to promote an annual street collection, and to advocate the principles of the League by means of a monthly gazette.

Sir J. Arnott's generous donation of £1,000 has been distributed among the following Cork City Hospitals, viz., South Infirmary, £250; North Infirmary, £200; Fever Hospital, £150; Mercy Hospital and Women and Children's Hospital, £100 each; Blind Asylum, £75; Lying-in Hospital, £50, and some other institutions £25 each.

The Chairman and Managing Committee of the Hull Infirmary have received a communication from Messrs. Reckitt of their intention of presenting the Withernsea Hotel and grounds to the hospital as a convalescent home. The authorities, in acknowledging the generous gift, express their sense of the benefit the donation will confer on the sick poor of the neighbourhood.

The Trustees of the Manchester Royal Infirmary have decided that no extension on the present site of that building shall take place. The ballot papers showed a majority of 140 against the proposal of the Board of Management, but only a little over half of those authorized to vote recorded their votes. In consequence of this decision, the Infirmary will be carried on in the accustomed way.

The total number of patients (34,168) treated during the year at the Leeds Public Dispensary shows an increase of 1,500 on that of last year, notwithstanding that there has been no such widespread epidemic of influenza as the committee had to report at their annual meeting in 1891, nor prevalence of any infectious disease; 3,023 patients have been visited during the year at their own homes, entailing 15,997 visits.

The number of patients of all classes treated during the year at the Lewes Dispensary and Victoria Hospital has been 1,481, which, with 136 remaining on the books the previous year, make a total of 1,617. During the year much has been done by the directors in improving the accommodation for the staff, ventilation of the wards and drainage, and necessary additions have also been made to the fittings and furniture.

A site, fourteen acres in extent, has been purchased for the new hospital at Halifax. The subscriptions already promised, along with the proceeds of the sale of the old infirmary, will, it is believed, be sufficient to cover the cost of the new hospital. The plan provides for from 130 to 150 beds; it is proposed to build an administrative department on a scale sufficient for a hospital of 300 beds should funds be available for that extension.

During the past year the following legacies have been received by the Committee of the Salisbury Infirmary: £1,2[illegible]0 from Miss Chafyn Grove, to endow the "Julia" cot; £100 from Mrs Mary Rawes, £[illegible]00 from Mrs. E. H. Carter, £[illegible]00 from Miss E. F. Frizell, £100 from Mrs. Cooper, £100 from Mr. James E. Nightingale, £300 from Mrs. A. H. Wyndham, and £50 from Mr. Peter Berry, the last six being all free of duty and costs. The Committee have also received for the thirteenth year the liberal donation of £30 from Mr. Rawlence, for the maintenance of a cot in the Children's Ward, "In Memoriam of Anges and Ellie Rawlence."

### THE STUDENT OF MEDICINE.

[All communications for this Supplement should be addressed to The Editor of The Hospital, at 140, Strand, with the words, "Students' Column," written in the left hand top corner of the envelope.]

### VACANCIES.

The following vacancies are announced this week, the amount of salary in each case being given after the name of the institution:

### Resident Medical Officers.

Sussex County Hospital, Assistant House-Surgeon. Residence, board, washing—Salary £30 per annum.
General Hospital, Nottingham. A senior Resident Medical Officer. Elected for two years, and eligible for re-election for similar term. Board, residence, washing—Salary £120 for first year, with an addition of £10 a year up to £150.
Cheltenham General Hospital, Branch Dispensary, Resident Surgeon. Residence, coal, and gas—Salary £180 per annum.
Bethlem Hospital, Two resident clinical assistants.
Central London Ophthalmic Hospital, House Surgeon. Rooms, coal, and light.

### Non-Resident Medical Officers.

Ancoats Hospital, Manchester, Hon. Physician.

### PASS LISTS.

### Royal College of Physicians of London.

The following candidates, having conformed to the bye-laws and regulations, and passed the required examinations, were at the meeting of the College on July 28th admitted Licentiates of the College: S. Ali, Lahore Medical School; J. Atcherley, Leeds; J. C. Baker, St. Bartholomew's; H. A. Ballance, University College; C. W. R. Banham, London; H. C. Barnes, London; *H. E. Blake, University College; E. W. Bliss, Birmingham; W. E. Bond, St. Bartholomew's; R. A. Bowie, McGill College, Montreal; R. W. Brimscombe, St. Mary's and Birmingham; H. C. Brown, Birmingham; S. A. Bull, Westminster; P. V. Bunch, University College; C. Buttar, St. Bartholomew's; T. Carwardine, Middlesex; G. W. Cazalet, London; B. Charles, St. Bartholomew's; G. Cross, St. Thomas's; M. Cutliffe, St. Bartholomew's; G. F. Darker, St. Thomas's; R. Davies, Edinburgh; C. A. Dixon, Leeds; F. Dove, London; R. H. W. Dunderdale, Liverpool; E. T. P. Eames, London; F. W. Edwards, St. Thomas's; J. S. Fowler, Edinburgh; H. St. J. Fraser, Guy's; H. C. French, King's College; R. J. Fyffe, London; A. Gaskell, University College; A. S. Gedge, St. Thomas's; H. E. Girdlestone, St. Thomas's; R. J. Gladstone, Aberdeen and Middlesex; T. H. J. C. Goodwin, St. Mary's; A. Graydon, St. Thomas's; R. C. Griffith, Toronto; S. A. E. Griffiths, Middlesex; J. Hague, Manchester; E. M. Hainworth, St. Thomas's; T. R. Hamlen-Williams, Middlesex; F. D. Harris, St. Mary's; E. B. Hartnell, Bristol and Guy's; J. R. Harvey, Guy's; A. D. Heath, University College; E. S. Hemsted, St. Mary's R. Henry, St. Thomas's; M. L. Hepburn, St. Bartholomew's; J. D. Hessey, Middlesex; R. H. Hogg, University of New Zealand; C. S. Jaffé, St. Thomas's; R. B. James, Birmingham; J. E. M. Jenkins, Charing Cross; J. Jones, University College; S. Kent, Cambridge and St. Bartholomew; St. J. B. Killery, St. Bartholomew's; M. H. Knapp, Cork and St. Mary's; M. C. Langford, London; J. P. Lightfoot, King's College; W. C. Loos, University College; C. V. McCormack, Liverpool; G. Mackeson, Guy's; A. E. Milner, Bristol; A. R. O. Milton, St. Thomas's; G. F. Morley, St. Georges's; J. L. Morton, St. Mary's; C. B. T. Musgrave, University College; G. F. M. Nellen, Middlesex; H. W. Newton, Middlesex; J. M. Nicol, Leeds; A. J. Ortlepp, St. Mary's; F. A. Osborn, Guy's; A. G. Parrott, London; *F. Perceval, St. George's; S. H. Perry, King's College; W. A. Perry, University College; W. L. Pethybridge, St. Bartholomew's; R. N. U. Pickering; St. Bartholomew's; E. Posnett, Leeds; W. Potter, St. Thomas's; W. A. Powell, Charing Cross; M. Randall, University College; H. F. Ransome, Manchester; E. G. Renny, St. Thomas's; C. H. Rock, St. Thomas's; J. R. Russell, King's College; J. L. Sawers, University College; J. G. Scheurer, London; F. C. Scotson, Manchester; E. W. Selby, University College; W. Selby, St. Bartholomew's; A. E. Shaw, St. Mary's; J. H. Sims, St. Thomas's; E. Smith, St. Thomas's; N. I. Smith, Middlesex; W. R. Smith, King's College; C. G. Spencer, University College; J. H. Sproat, Birmingham; R. E. P. Squibbs, Middlesex; H. E. Staddon, St. Thomas's; S. L. J. Steggall, Charing Cross; J. A. Stewart, Charing Cross; A. Strange, London; W. G. Sutcliffe, St. Thomas's; M. Swabey, St. Bartholomew's; H. M. Sylvester, St. Bartholomew's; G. Templeton, Edinburgh; R. S. Thomas, Middlesex; H. H. Tipping, Birmingham; N. P. F. Toller, St. Thomas's; R. C. Tweedy, St. Bartholomew's; W. J. N. Vincent, Durham and London; M. H. Vinrace, Birmingham and University College; F. Wall, Ceylon and Middlesex; R. A. Walter, St. Bartholomew's; A. R. Walters, Cambridge and London; F. W. Walters, London; W. Watkins, London; F. W. Wesley, University College; W. G. West, St. Bartholomew's; E. W. Wheatcroft, Guy's; J. A. H. White, Birmingham; G. C. W. Williams, St. Thomas's; H. J. E. H. Williams, Middlesex; H. W. S. Williams, University College and Liverpool; E. H. Willock, St. Thomas's; J. W. Willson, Charing Cross; F. N. Windsor, Manchester; L. A. Winter, St. Bartholomew's; F. E. Withers, St. Bartholomew's; S. F. Wright, St. Thomas's; W. C. H. Wroughton, St. Thomas's; A. T. Wysard, St. Thomas's.

* Candidates who have not presented themselves under the regulation of the Examining Board.

### THE STUDENT OF MEDICINE—(*continued*).

### University of Edinburgh.

The following are the official lists of candidates who passed the second examinations at Edinburgh University last month:—

SECOND PROFESSIONAL EXAMINATION.—C. H. Adamson, A. C. Ainslie (M.A.), R. Y. Anderson. Harry Armistead. Ayatullah, William Bannerman, F. W. Barton, R. W. Blair, R. St. G. S. Bond, D. J. S. Burt, C. H. H. Cazalet, W. R. Center, D. N. Chatterjee, W. B. Clarke, D. M. Conacher, H. C. Corfield, J. R. Crease, E. A. Dent, E. P. Dickin. G. A. Dickson, P. G. Edgar, Henry Ferguson, Arthur Foster, G. E. Gabites, W. H. Gaunt, J. R. Gilmour (with distinction), Lachlan Grant, James Gray, D. A. Greaves, F. H. Hardman, W. B. Hetherington, J. E. Hewlett, John Hume, T. J. Jehu, Evan Jones, W. H. Jones, John Kippax (with distinction), G. C. Laing, John Lawrie, J. L. Leadbetter, J. A. Lee, Donald MacDonald, Andrew M'Neil (B.A.), P. W. MacVean, W. S. Malcolm, J. M. Menzies, T. B. Moore (B.A.), J. M. Morris (M.A.), David Murray, C. J. Murray, F. B. Oliphant, E. L. Owen, J. M. Pereira, E. L. Phillips, G. B. Pieterson, G. R. Plante, S. W. Prowse (B.A.), J. H. Reynolds, C. H. Ridley, F. W. H. Robson, J. E. Rogers, A. A. Ross, C. E. Salt, Henry Scott, W. E. Scott-Moncrieff, J. H. Seon, P. J. Sharp, T. D. S. Shaw, T. R. Sibbald, W. E. Smith, John Sorley (M.A.), A. P. Steavenson, J. W. Sutcliffe, Thomas Taylor, H. W. Vaughan-Williams, J. G. Walker, J. W. T. Walker, E. E. Waters, J. M. Wishart, J. J. H. Wood, R. J. T. Wright (M.A.), C. W. C. Branch, W. H. Coupland.

#### THE DEGREE OF DOCTOR OF MEDICINE.

R. D. R. Allison, Scotland, M.D., C.M. (with second-class honours), 1889; G. T. Beatton, Scotland, M.B., C.M., 1887; * G. S. Brock, Scotland, M.B., C.M. (with second-class honours), 1880; † A. W. Campbell, Australia, M.B., C.M., 1889; † H. M. Chasseaud, Asia Minor, M.B., C.M., 1890; ‡ H. M. Clark, India, M.B., C.M., 1881; E. W. Clarke, England (B.Sc.), M.B., C.M., 1885; ‡ F. G. Clemow, England, M.B., C.M. (with second-class honours), 1885; J. O. Closs, Scotland, M.B., C.M., 1882; ‡ G. M. Cullen, Scotland. M.B., C.M., 1889; J. A. Dick, Australia (B.A.), M.B., C.M., 1890; W. Doig, Scotland, M.B., C.M., 1881; † E. H. Ezard, England, M.B., C.M., 1887; ‡ R. J. Fox, India, M.B., C.M., 1887; E. V. Gibson, England, M.B., C.M., 1890; * A. L. Gillespie, Scotland, M.B., C.M., 1888; ‡ W. W. Hall, India, M.B., C.M., 1884; ‡ A. H. Hallen, England, M.B., C.M., 1889; ‡ J. G. Havelock, England, M.B., C.M., 1887; ‡ J. H. Keay, Scotland (M.A.), M.B., C.M., 1876; J. T. Kitchin, England, M.B., C.M., 1887; ‡ G. Leslie, Scotland, M.B., C.M. (with second-class honours), 1881; ‡ W. Lockwood, England, M.B., C.M., 1888; ‡ A. J. MacGregor, Scotland. M.B., C.M., 1888; N. J. M'Kie, England, M.B., C.M., 1887; ‡ C. W. S. Magrath, England, M.B., C.M., 1880; C. J. L. Mansel, England, M.B., C.M., 1889; E. F. Maynard, England, M.B., C.M., 1889; R. T. Meadows, Canada, M.B., C.M., 1885; W. G. Mitchell, Scotland (M.A.), M.B., C.M., 1885; B. G. Morison, India, M.B., C.M. (with first-class honours), 1878; A. W. Munro, Scotland, M.B., C.M. 1883; W. Murray, Scotland, M.B., C.M., 1879; A. B. Northcote, England, M.B., C.M., 1883; J. W. Pare, England, M.B., C.M., 1885; W. M. Parham. England, M.B., C.M., 1889; ‡J. Playfair, Scotland, M.B., 1872; †T. D. Poole, England, M.B., C.M., 1889; R. J. Pope, Australia (B.A.), M.B., C.M., 1890; †J. Russell, Scotland (M.A.), M.B., C.M., 1888; C. B. B. Savory, England, M.B., C.M., 1888; ‡J. B. Simpson, Scotland (M.A.), M.B., C.M., 1887; G. A. V. Someren, Scotland, M.B., C.M., 1882; ‡G. H. H. Symonds, England, M.B., C.M., 1884; W. E. Thomas, Wales, M.B., C.M., 1885; I. Thompson, England, M.B., C.M. (with second-class honours), 1888; J. C. Thomson, Scotland (M.A.), M.B., C.M. (with second-class honours), 1888; G. Thornton, England, M.B., C.M., 1890; ‡A. Turner, England, M.B., C.M., 1885; *W. A. Turner, Scotland, M.B., C.M. (with first-class honours), 1887; T. Walcot, England, M.B., C.M., 1887; ‡E. Walker, England, M.B., C.M. (with first-class honours), 1884; T. H. Ward, England, M.B., C.M. (with first-class honours), 1887; W. M. C. Watson, Scotland, M.B., C.M., 1888; S. R. Webb, England, M.B., C.M., 1890; W. H. Weston, England, M.B., C.M., 1881; *A. J. Whiting, England, M.B., C.M., 1889; *T. S. Wilson, England (B.Sc.), M.B., C.M. (with second-class honours), 1883; A. B. Winder, England, M.B., C.M., 1884; ‡R. Wise, Scotland, M.B., C.M., 1888.

* Those who have obtained gold medals for their dissertations.
† Deemed worthy of competing for the dissertation gold medals.
‡ Commended for their dissertations.

### Army Medical Pass List.

The following is a list of the candidates for Her Majesty's Indian Medical Service who were successful at the competitive examination held at Burlington House on August 8th, 1892, and following days:—

| | Marks. | | Marks. |
|---|---|---|---|
| Drake-Brockman, V. G. | 2,850 | Ogilvie, W. H. | 2,490 |
| Milne, C. | 2,745 | Heard, R. | 2,405 |
| Prall, C. B. | 2,720 | Bourke, J. J. | 2,385 |
| Hunter, G. Y. C. | 2,640 | Bidie, G. | 2,305 |
| Young, W. | 2,620 | Orr, W. H. | 2,230 |
| Macleod, J. N. | 2,615 | More, P. St. C. | 2,200 |
| Chatterton, B. R. | 2,600 | Parry, E. R. | 2,195 |
| Williams, C. E. | 2,580 | Morton, J. P. | 2,185 |
| Langston, T. A. O. | 2,490 | | |

Twenty-nine candidates competed for seventeen appointments; twenty-one were reported qualified.

